# Nitric Oxide Synthase 2 Promoter Polymorphism Is a Risk Factor for Allergic Asthma in Children

**DOI:** 10.3390/medicina57121341

**Published:** 2021-12-08

**Authors:** Joanna Nowakowska, Paulina Sobkowiak, Anna Bręborowicz, Magdalena Mrówczyńska, Irena Wojsyk-Banaszak, Aleksandra Szczepankiewicz

**Affiliations:** 1 Molecular and Cell Biology Unit, Poznan University of Medical Sciences, 60-572 Poznan, Poland; alszczep@gmail.com; 2 Department of Pediatric Pulmonology, Allergy and Clinical Immunology, Poznan University of Medical Sciences, 60-572 Poznan, Poland; paulina-25@tlen.pl (P.S.); abreborowicz@wp.pl (A.B.); magmrow@poczta.fm (M.M.); iwojsyk@ump.edu.pl (I.W.-B.)

**Keywords:** NO synthases, *NOS2*, childhood asthma, genetic polymorphism

## Abstract

*Background and Objectives*: In paediatric population, atopic asthma is associated with increased eosinophil counts in patients, that correlate with the airway inflammation measured by the concentration of nitric oxide in exhaled air (FeNO). As the FeNO level is a biomarker of atopic asthma, we assumed that polymorphisms in nitric synthases genes may represent a risk factor for asthma development. The purpose of this study was to analyse the association of *NOS* genetic variants with childhood asthma in the Polish population. *Materials and methods*: In study we included 443 children—220 patients diagnosed with atopic asthma and 223 healthy control subjects. We have genotyped 4 single nucleotide polymorphisms (SNP) from 3 genes involved in the nitric oxide synthesis (*NOS1*, *NOS2* and *NOS3*). All analyses were performed using polymerase chain reaction with restriction fragments length polymorphism (PCR-RFLP). *Results:* We observed significant differences between cases and controls in SNP rs10459953 in *NOS2* gene, considering both genotypes (*p* = 0.001) and alleles (*p* = 0.0006). The other analyzed polymorphisms did not show association with disease. *Conclusions*: According to our results, 5′UTR variant within *NOS2* isoform may have an impact of asthma susceptibility in the population of Polish children. Further functional studies are required to understand the role of iNOS polymorphism in *NOS2* translation and to consider it as a novel risk factor in childhood asthma. The next step would be to apply this knowledge to improve diagnosis and develop novel personalized asthma therapies.

## 1. Introduction

Asthma is a heterogeneous disease characterized by chronic airway inflammation, variable airflow limitation and airway hyperresponsiveness. Type of airway inflammation differs between asthmatic patients, but in childhood asthma up to 80% of patients demonstrates T helper cells subtype 2 (Th2)-mediated allergic inflammation with overexpression of Th2 cytokines (e.g., IL-4, IL-5 and IL-13), increased IgE production and enhanced recruitment of eosinophils to the site of inflammation in the airways [1]. In these cases, asthma is frequently associated with atopic dermatitis and rhinitis [2,3]. Increased eosinophil counts correlate with the fractional concentration of nitric oxide in exhaled air (fraction of exhaled NO; FeNO) and this parameter is an useful and non-invasive biomarker of airway inflammation severity [4]. Previous studies have shown increased levels of FeNO in asthmatic individuals that associated with eosinophilic inflammation in the airways [5,6]. Moreover, several previous papers reported increased FeNO concentration in paediatric and adult asthmatic patients [7,8,9], also in particularly in relation to atopic phenotype [10,11]. In a 3-year-follow-up study elevated FeNO was associated with an increased risk for new-onset asthma in schoolchildren, thus making this parameter a potential asthma predictor [12]. Despite many years of research, as well as awareness of the relationship of FeNO with asthma exacerbations, its clinical value in the population of children is still not fully understood [13]. Nitric oxide (NO) is formed by conversion of L-arginine to L-citrulline in the presence of one of three distinct isoforms of NO synthases (NOSs): a neuronal isoform (nNOS or NOS1), an endothelial isoform (eNOS or NOS3) and inducible NOS (iNOS or NOS2). The NOS1 and 3 each generate small amount of NO in the lungs whereas NOS2 generates the highest amounts of NO during inflammation [14]. These isoforms are encoded by three different genes (*NOS1, NOS2* and *NOS3*, respectively). *NOS1* is expressed in human airway nerves, *NOS3* in endothelial cells of the pulmonary circulation and *NOS2* in T cells, macrophages, epithelial cells, mast cells, eosinophils and neutrophils in response to inflammatory stimuli such as cytokines, oxidants, viral infections and allergen exposure [15,16]. Previous genetic studies showed that *NOS* polymorphisms were associated with asthma and FeNO levels in adult and paediatric populations worldwide, including large cohort study of over 2000 white children of Hispanic and non-Hispanic origin from California [17]. However, no previous study analysed the association between asthma and NOS genetic variants in paediatric asthmatics of Central European origin. Therefore, the aim of our study was to determine whether the previously reported associations of NOS variants with asthma are also observed in Polish asthmatic children. In this case-control study we investigated a possible association of the single nucleotide polymorphisms (SNPs) in all three NOS genes with IgE-mediated childhood asthma and FeNO levels.

## 2. Materials and Methods

In this case-control study, we included 443 Polish children: 220 patients diagnosed with atopic asthma and 223 healthy control subjects. Asthma diagnosis was made according to Global Initiative for Asthma (GINA) guidelines and included lung function test, fractionated exhaled nitric oxide (FeNO) measurement and atopic parameters (serum total IgE level, skin prick tests with common inhaled allergens). The detailed clinical description of the performed tests was previously described by Szczepankiewicz et al. [18].

Control group: Control group included 223 healthy subjects from the same geographic region. In the control group, clinical examination excluded current and past asthma or allergy symptoms, we also confirmed normal spirometry and normal FeNO concentration (<20 ppb) in these children.

Genotyping: We have genotyped 4 SNPs from 3 genes involved in the nitric oxide synthesis (*NOS1, NOS2* and *NOS3*). The SNP selection was based on at least two of the following criteria: functionality demonstrated in the previous experimental studies, minor allele frequency above 0.05 or previous association with asthma or atopy. DNA was extracted from 10 mL of EDTA anticoagulated whole blood using the salting out method presented by Miller et al. [19] or from the saliva using Oragene DNA isolation system (DNA Genotek, Ottawa, ON, Canada, ). Genotyping of polymorphisms was performed using PCR-RFLP (polymerase chain reaction with restriction fragments length polymorphism) method. PCR reactions with specific primers were performed in MasterCycler (Eppendorf, Hamburg, Germany) thermal cycler. PCR products for each sample were then digested overnight (37 °C for Eco72I, FspBI and MboI, 65 °C for FspBI and TasI) with 0.5 U of appropriate restriction endonuclease. After RFLP, the digested products were resolved in agarose gel to identify the genotypes. Undigested PCR products for analyzed polymorphisms were re-digested to confirm the results. Primer sequences, PCR product size, restriction endonucleases used for genotyping and band size for particular alleles were shown in Table 1. For each reaction plate genomic control DNA samples and non-template controls were included. To check for RFLP genotyping accuracy, 25% of randomly chosen samples from both groups for each SNP were repeatedly analyzed. Genotyping was performed without knowing the clinical status of the subjects.

Statistical analysis: Pearson’s Chi-square (χ^2^) test and Fisher’s exact test were used to test differences in the genotypic and allelic (respectively) distribution in case control analysis. Stepwise logistic regression analysis was performed using disease status (case-control) as dichotomous dependent variable and the following predictors included in the model: analyzed polymorphisms, exhaled nitric oxide level, age and gender. Calculations were performed using Statistica version 10.0 software. Concordance with Hardy–Weinberg law was performed using a calculator developed by Court lab (Court M 2008). Higher-order gene-gene interactions were analyzed using genetic model-free multifactor dimensionality reduction (MDR) approach (v.2.0 beta 8.3) [20] using criteria described in the previous study [18].

## 3. Results

The group of patients and control subjects were similar in age and gender (*p* > 0.05). The asthmatic children showed significantly decreased lung function and increased FeNO levels (*p* < 0.001). The results were shown in Table 2.

### 3.1. Hardy–Weinberg Equilibrium Test

All studied polymorphisms were tested for concordance with the Hardy–Weinberg equilibrium law (HWE). No deviation was observed for asthma patients and control group (*p* > 0.05).

### 3.2. Single Marker Association Results

Genotype distribution and allele frequency were significantly different for rs10459953 polymorphism in *NOS2* gene and TT genotype and T allele were significantly more frequent in the asthmatic patients (*p* = 0.001 and *p* = 0.0006, respectively). The other analyzed polymorphisms did not show association with the disease. The results of association analysis were presented in Table 3. The analysis stratified by gender confirmed strong association of rs10459953 polymorphism in *NOS2* gene with asthma showing significantly higher frequency of TT genotype and T allele among asthmatic boys (*p* = 0.00002 for genotypes, *p* = 0.000001 for alleles), but not in girls (*p* > 0.05) (data not shown).

### 3.3. Gene × Gene Interaction Analysis

We found a significant epistatic interaction between two variants of *NOS2* gene (rs2297518 and rs10459953, 2-locus model) and childhood asthma risk. The testing balanced accuracy was 63.3% and the cross-validation consistency was 80% (*p* = 0.025 after 1000 permutations). One of these polymorphisms (C/T in *NOS2* gene) showed a significant association with the risk of asthma in a single marker analysis. We did not observe any epistatic interaction between NOS polymorphisms and exhaled NO level. Results of interaction analysis of analyzed SNPs for each comparison were summarized in Table 4.

## 4. Discussion

Our study shows an association between rs10459953 in *NOS2* gene and childhood asthma, which was the first to explore it in Polish population. Only a few studies have analyzed this polymorphism in the context of various diseases, such as depression [21], benign prostatic hyperplasia [22], diabetes mellitus [23], male infertility [24] and stroke [25], but none of them investigated it in the context of asthma. Some studies considered *NOS2* polymorphisms in various populations. Salam et al. analysed childhood asthma in US population, stratified by origin (Hispanic versus non—Hispanic), and identified complex sets of haplotypes in the *NOS2* promoter coding regions and these haplotypes were associated with FeNO in children with and without asthma [17]. In turn, Bouzigon et al. found significant associations of FeNO levels with two SNPs in *NOS3* (rs2853796 and rs1549758) and one SNP in *NOS2* (rs6505510) in non-asthmatic adults. Considering asthmatic subjects, a single association was detected between FeNO levels and one SNP in *NOS3* (rs743507) [26]. In a Japanese population, the number of CCTTT repeats in the *NOS2* promoter region appeared to be significantly associated with FeNO levels in adults, but only before the treatment with inhaled corticosteroids. The same study researched also seven SNPs in this gene, none of them were related to FeNO levels [27]. Dahgam et al. analysed 49 SNPs in *NOS2, NOS1* and *NOS3* in Swedish adult general population, considered two in *NOS2* (rs9901734 and rs3729508) and one in *NOS3* (rs7830) as independently associated with FeNO levels. In rs7830, the relation depended on asthma status, suggesting that *NOS3* genetic variation plays the most important role among all nitric oxide synthases in asthmatic adults [28]. In our study, we found that *NOS2* variant located in the 5′UTR region was associated with childhood asthma in the Polish population. In addition, result of epistatic analysis showed only significant epistatic effect between two variants within *NOS2*, suggesting that this combination affects asthma susceptibility, but not FeNO levels. This is the first analysis showing that 5′UTR variant in *NOS2* gene may be associated with the risk of childhood asthma. Although the functional effect of this polymorphism on *NOS2* posttranscriptional regulation has not been determined yet, the recent study by Sample et al. [29] showed that 5′UTR variants may affect 5 cap structure and ribosome loading and thus affect translation.

The results of *NOS1* and *NOS3* association studies were inconsistent depending on the studied population. For example, *NOS1* polymorphism (AAT repeats in intron 20) was associated with higher FeNO concentration in adult asthmatics of Caucasian origin [30], but later studies showed a relationship between *NOS1* variants and FeNO also in healthy population and suggested they were associated with FeNO levels in women. Such association was not observed for *NOS3* variants [31]. Some *NOS1* and *NOS3* polymorphisms studied in various populations showed a positive correlation with atopy [32], total IgE concentration [33,34] or sensitization to inhaled allergens (e.g., Dermatophagoides pteronyssinus) and a possible relationship with the risk of asthma [35]. The polymorphism in *NOS1* gene correlated with the physician-diagnosed asthma and the risk of severe exacerbation in children, as demonstrated by Duckworth et al., but such relationship was not observed in regard to the polymorphism in *NOS3* gene [36].

As genetic variants within NOS genes may influence NO levels in asthmatic airways, they may also influence allergic response and secretion of Th2 cytokines. The most important cytokines associated with allergic asthma are IL-4, IL-13 and IL-5, that are related to the activity of immune cells such as T cells, B cells producing specific IgE antibodies, eosinophils and mast cells [37]. The secretion of IL-5 results from an increased number of eosinophils recruited in the process of allergic inflammation, while enhanced release of IL-4 and IL-13 from Th2 cells, affect epithelial cells, thus contributing to increased fractional exhaled nitric oxide levels in the asthmatic airways [38]. Our previous study showed the association of IL-4, its receptor IL4Rα and IL-13 polymorphisms with asthma in the same pediatric cohort that was analyzed in this study [39]. Therefore, these studies confirm that Th2 cytokines are associated with allergic asthma in our pediatric population.

## 5. Conclusions

Taken together, our results suggest, that 5′UTR variant within inducible NOS isoform may affect susceptibility of childhood asthma in Polish population. However, further functional studies are required to elucidate if this variant affects *NOS2* translation and thus may represent of novel risk factor in the allergic asthma. The next step would be to apply this knowledge to improve diagnosis and develop novel personalized asthma therapies.

## Figures and Tables

**Table 1:** The sequences of PCR primers and restriction enzymes used in genotyping analysis.

Gene	SNP	Primer Sequences	Product Size (bp)	Restriction Enzyme	Alleles (bp)
NOS1	C5266T rs2682826	F: 5-ACTCCTTGAGTTTTCCTGCTGCGATG-3 R: 5-CCATGTTCCAGTGGTTTCATGCACAC-3	128	Eco72I	T: 128 C: 100, 28
NOS2	-1659C/T rs10459953	F: 5′-TGCTGCAGGTATAGCCAGAAT-3′ R: 5′-ATGGAGGGATGGTATGGTGCTGAT-3′	159	FspBI	C: 160 T: 129, 31
	608C/T rs2297518	F: 5′-ATCCCCTGAACCCAGACTTT-3′ R: 5′-GGCCAGGTTTCCAGAAGAA-3′	198	TasI	C: 175, 113 T: 142, 113
NOS3	G894T rs1799983	F: 5′-AAGGCAGGAGACAGTGGATG-3′ R: 5′-CAGTCAATCCCTTTGGTGCT-3′	246	MboI	G: 246 T: 158, 88

SNP—single nucleotide polymorphism; Set of F—forward and R—reverse primers sequences, bp—base pairs.

**Table 2:** Clinical description of analyzed population.

Parameter	Patients Group (*n* = 220)	Control Group (*n* = 223)
Gender, male (%)	59%	48%
Age, years (mean ± SD)	11.45 ± 3.56	12.14 ± 3.31
Asthma severity		-
Mild, *n* (%)	29 (13.2)	-
Moderate, *n* (%)	121 (55.0)	-
Severe, *n* (%)	70 (31.8)	-
Positive SPT (%)	68.2	-
FEV1/FVC pred (mean ± SD)	88.5 ± 12.5	104.35 ± 8.18
FEV1% pred (mean ± SD)	85.3 ± 15.1	106.15 ± 12.1
FVC% pred (mean ± SD)	89.9 ± 15.1	100.5 ± 10.9
PEF% pred (mean ± SD)	81.3 ± 15.8	90.3 ± 12.9
FeNO (ppb) (mean ± SD)	28.6 ± 34.3	16.6 ± 14.4
IgE (IU/mL) (mean ± SD)	257.5 ± 326.7	-

FEV1—forced expiratory volume in the first second, FVC—forced vital capacity, PEF—peak expiratory flow, FeNO—fraction of exhaled NO.

**Table 3:** The association analysis for analyzed polymorphisms between asthmatic patients and the control group.

Gene	Polymorphism			Asthma (%)	Control (%)	*p* Value
NOS1	C5266T rs2682826	Genotypes	CC CT TT	57.3 37.3 5.45	58.6 33.3 8.1	0.659
		Alleles	C T	24.1 75.9	24.7 75.3	0.912
NOS2	-1659C/T rs10459953	Genotypes	CC CT TT	67.2 31.3 1.5	86.8 12.4 0.8	0.001 *
		Alleles	C T	82.8 17.2	93.0 7.0	0.0006 *
	608C/T rs2297518	Genotypes	CC CT TT	70.8 26.9 2.3	68.5 29.7 1.8	0.866
		Alleles	C T	84.2 15.8	84.1 15.9	0.532
NOS3	G894T rs1799983	Genotypes	GG GT TT	54.5 40.0 5.5	51.2 42.2 6.6	0.855
		Alleles	G T	74.5 25.5	72.3 27.7	0.590

* Indicates significance.

**Table 4:** The results of multifactor dimensionality reduction analysis for epistatic interaction between analyzed variants in NOS genes.

Model	Loci Combination	Testing Balanced Accuracy (%)	Cross-Validation Consistency	*p*-Value
Asthma				
2-locus	NOS2 (608) and NOS2 (C/T)	62.3	8	0.025 *
3-locus	NOS1, NOS2 (608) and NOS2 (C/T)	58.4	6	0.201
4-locus	NOS1, NOS3, NOS2 (608) and NOS2 (C/T)	53.7	10	0.640
Increased FeNO				
2-locus	NOS2 608 and NOS3	44.9	6	0.961
3-locus	NOS1, NOS2 608 and NOS3	51.1	8	0.763
4-locus	NOS1, NOS3, NOS2 (608) and NOS2 (C/T)	55.7	10	0.476

* Indicates significance.
